# Differences in Characteristics and Clinical Outcomes Among Hispanic/Latino Men and Women Receiving HIV Medical Care — United States, 2013–2014

**DOI:** 10.15585/mmwr.mm6740a2

**Published:** 2018-10-12

**Authors:** Ruth E. Luna-Gierke, R. Luke Shouse, Qingwei Luo, Emma Frazier, Guangnan Chen, Linda Beer

**Affiliations:** ^1^Division of HIV/AIDS Prevention, National Center for HIV/AIDS, Viral Hepatitis, STD, and TB Prevention, CDC; ^2^ICF International, Fairfax, Virginia.

The prevalence of diagnosed human immunodeficiency virus (HIV) infection among Hispanics/Latinos in the United States is approximately twice that of non-Hispanic whites (*1*). Barriers to, and experiences with, medical care have been found to vary by sex (*2*). Describing characteristics of Hispanics/Latinos in care by sex can help identify disparities and inform delivery of tailored services to this underserved population. Data from the 2013 and 2014 cycles of the Medical Monitoring Project (MMP) were analyzed to describe demographic, behavioral, and clinical characteristics among Hispanics/Latinos by sex. MMP is an annual cross-sectional, nationally representative surveillance system that, during 2013–2014, collected information about behaviors, medical care, and clinical outcomes among adults receiving outpatient HIV care. Hispanic/Latina women were significantly more likely than were men to live in poverty (78% versus 54%), report not speaking English well (38% versus 21%), and receive interpreter (27% versus 16%), transportation (35% versus 21%), and meal (44% versus 26%) services. There were no significant differences between Hispanic/Latino women and men in prescription of antiretroviral therapy (ART) (95% versus 96%) or sustained viral suppression (68% versus 73%). Although women faced greater socioeconomic and language-related challenges, the clinical outcomes among Hispanic/Latina women were similar to those among men, perhaps reflecting their higher use of ancillary services. Levels of viral suppression for Hispanics/Latinos are lower than those found among non-Hispanic whites (*3*) and lower than the national prevention goal of at least 80% of persons with diagnosed HIV infection. Providers should be cognizant of the challenges faced by Hispanics/Latinos with HIV infection in care and provide referrals to needed ancillary services.

MMP data were collected annually during 2013–2014 using three consecutive sampling stages (states and territories, outpatient HIV facilities, and patients), and response rates for the two cycle-years of data that were included in the analysis were 100% (states and territories), 85%–86% (outpatient HIV facilities) and 55%–56% (patients). Data were collected using face-to-face or telephone interviews and medical record abstraction from June 2013 through May 2015.

The analysis included 1,774 men and 577 women who self-identified as Hispanic or Latino, regardless of race. Data were self-reported from the interview and abstracted from the respondent’s medical record. Data were weighted based on known probabilities of selection and adjusted for facility and patient non-response. Rao-Scott chi-square tests were used to assess differences by sex; p-values <0.05 were considered statistically significant. Selected sociodemographic and behavioral variables, use of ancillary services, and clinical outcomes are presented by sex. All analyses accounted for the complex sample design and weights.

Women were significantly more likely than were men to live in poverty (78% versus 54%), live in a household with ≥1 dependents aged <18 years (66% versus 37%), have public insurance coverage (72% versus 54%) and, among those living outside of Puerto Rico, report not speaking English well (38% versus 21%) (Table 1). Compared with men, women were less likely to have more than a high school education (28% versus 47%), be employed (29% versus 48%), have any private insurance (14% versus 22%), and have been born outside the United States (36% versus 45%). Women most often reported their country or region of origin in the Caribbean (including Puerto Rico) (38%), followed by the mainland United States (33%). Men most often reported the mainland United States as their country or region of origin (36%), followed by Mexico and Central America (32%) (Table 1). Women were also less likely than were men to report using stimulants (3% versus 10%), non-injection drugs (8% versus 23%), injection drugs (0.4% versus 3%), or any opioids (0.8% versus 3%) (Table 2). Women were more likely than men to receive interpreter (27% versus 16%), transportation (35% versus 21%), and meal services (44% versus 26%). Women did not report a greater unmet need for these services than did men. Among women and men, prescription of antiretroviral therapy (95% versus 96%) and prevalence of sustained viral suppression (68% versus 73%) did not significantly differ.

**TABLE 1:** Selected characteristics of Hispanics/Latinos receiving medical care for diagnosed HIV infection, by sex — United States, 2013–2014*

Characteristic	Total (N = 2,351)		Men (N = 1,774)		Women (N = 577)		Rao-Scott chi-square p-value comparing men and women
	No.	% (95% CI)^†^	No.	% (95% CI)^†^	No.	% (95% CI)^†^	
**Educational attainment**							
<High school	741	33.5 (29.4–37.7)	487	29.2 (25.4–33.0)	254	48.0 (38.5–57.4)	<0.0001
High school diploma or GED	571	24.1 (22.2–26.1)	435	24.3 (21.9–26.7)	136	23.6 (20.0–27.2)	
>High School	1,038	42.3 (37.8–46.8)	852	46.5 (42.2–50.8)	186	28.4 (19.6–37.3)	
**Employment**							
Employed	1,028	43.2 (39.3–47.1)	860	47.5 (43.9–51.1)	168	28.8 (24.2–33.3)	<0.0001
Unemployed	415	19.6 (17.2–21.9)	322	20.0 (17.7–22.3)	93	18.2 (13.2–23.1)	
Other	908	37.3 (32.7–41.8)	592	32.5 (28.9–36.2)	316	53.1 (46.1–60.0)	
**Annual household income**							
<$19,999	1,642	73.3 (70.0–76.5)	1,174	69.9 (66.5–73.3)	468	84.0 (80.9–87.1)	<0.0001
$20,000–$39,999	411	18.4 (16.1–20.6)	343	20.4 (18.0–22.7)	68	12.1 (9.1–15.0)	
≥$40,000	179	8.4 (6.7–10.0)	153	9.8 (7.7–11.8)	26	3.9 (2.5–5.4)	
**No. of household dependents aged <18 years**							
0	565	54.7 (50.7–58.7)	449	63.2 (58.6–67.9)	116	34.1 (25.8–42.3)	<0.0001
1–2	374	37.0 (33.4–40.5)	204	29.9 (25.7–34.2)	170	53.9 (46.6–61.2)	
≥3	85	8.4 (6.6–10.1)	49	6.8 (4.8–8.8)	36	12.1 (7.8–16.3)	
**Household poverty level^§^**							
Above	891	40.7 (36.2–45.2)	766	46.5 (42.3–50.8)	125	22.2 (18.1–26.3)	<0.0001
At or below	1,340	59.3 (54.8–63.8)	904	53.5 (49.2–57.7)	436	77.8 (73.7–81.9)	
**Health coverage or coverage for medications**							
Any private insurance	454	20.4 (17.7–23.2)	375	22.3 (19.2–25.3)	79	14.3 (11.1–17.5)	<0.0001
Public insurance only	1,363	57.9 (50.7–65.0)	954	53.6 (47.4–59.8)	409	72.2 (63.8–80.6)	
Uninsured or Ryan White HIV/AIDS Program coverage only	481	21.7 (15.3–28.0)	411	24.1 (18.2–30.1)	70	13.5 (6.3–20.7)	
**Homeless^¶^**	155	7.2 (5.7–8.7)	129	7.9 (6.3–9.6)	26	4.6 (2.9–6.4)	0.0015
**HIV acquisition risk**							
MSM	1,031	44.4 (39.4–49.4)	1,031	57.8 (53.8–61.8)	N/A	N/A	N/A
IDU	189	7.9 (6.3–9.6)	146	7.9 (5.7–10.1)	43	7.9 (5.5–10.4)	N/A
MSM and IDU	55	2.3 (1.5–3.1)	55	3.0 (2.0–4.0)	N/A	N/A	N/A
Heterosexual contact	405	16.3 (12.4–20.1)	119	7.0 (5.3–8.8)	286	46.8 (38.2–55.4)	N/A
Other**	671	29.1 (26.7–31.5)	423	24.2 (21.8–26.6)	248	45.3 (37.4–53.2)	N/A
**Speaks English (mainland United States only)**							
Well/Very well	1,400	75.4 (72.5–78.3)	1,153	79.0 (76.2–81.9)	247	62.0 (56.1–67.8)	<0.0001
Not well/Not well at all	473	24.6 (21.7–27.5)	317	21.0 (18.1–23.8)	156	38.0 (32.2–43.9)	
**Foreign born**	982	43.2 (33.1–53.4)	786	45.3 (36.4–54.2)	196	36.4 (22.6–50.2)	0.0007
**Country or region of origin**							
Mainland United States	751	35.5 (28.3–42.8)	586	36.4 (29.7–43.0)	165	32.8 (22.7–42.9)	<0.0001
Mexico and Central America	706	29.9 (21.7–38.2)	568	31.6 (24.3–38.8)	138	24.6 (13.1–36.0)	
South America	142	6.8 (4.5–9.1)	118	7.5 (5.1–9.8)	24	4.6 (1.9–7.3)	
Caribbean (including Puerto Rico)	741	27.8 (11.7–43.8)	492	24.6 (10.6–38.6)	249	38.1 (16.9–59.3)	
**Median years of U.S. residence (range)^††^**	—	19.7 (0–62)	—	19.7 (0–62)	—	19.3 (0–59)	—

**Abbreviations:** CI = confidence interval; GED = General Educational Development; HIV = human immunodeficiency virus; IDU = injection drug user; MSM = men who have sex with men; N/A = not applicable.

* Numbers might not sum to total because of missing data. Percentages might not sum to 100 because of rounding. All estimates are based on self-report from interview except where otherwise noted. All estimates are based on the 12 months preceding interview except where otherwise noted.

**^†^** Percentages are weighted percentages. 95% confidence intervals incorporate weighted percentages.

^§^ Poverty guidelines as defined by the Department of Health and Human Services (HHS). https://aspe.hhs.gov/poverty/faq.cfm.

^¶^ Living on the street, in a shelter, in a single-room-occupancy hotel, or in a car.

** Includes hemophilia, blood transfusion, perinatal exposure, and risk factor not reported or not identified.

**^††^** Among persons who were foreign-born.

**TABLE 2:** Selected behaviors and clinical outcomes of Hispanics/Latinos receiving medical care for diagnosed HIV infection, by sex — United States, 2013–2014*

Behavior/Clinical outcome	Total (N = 2,351)		Men (N = 1,774)		Women (N = 577)		Rao-Scott chi-square p-value comparing men and women
	No.	% (95% CI)^†^	No.	% (95% CI)^†^	No.	% (95% CI)^†^	
**Meets criteria for depression, past 2 weeks**							
No	1,854	80.2 (78.4–81.9)	1,425	81.6 (79.8–83.4)	429	75.5 (71.9–79.1)	0.0058
Other depression	235	9.7 (8.2–11.1)	166	9.1 (7.7–10.6)	69	11.3 (8.6–14.0)	
Major depression	235	10.2 (8.6–11.7)	160	9.3 (7.7–10.8)	75	13.2 (9.7–16.7)	
**Substance use**							
Binge drinking in past 30 days	401	16.6 (14.7–18.6)	353	19.3 (16.9–21.6)	48	8.0 (4.9–11.1)	<0.0001
Non-IDU	423	19.5 (16.7–22.3)	380	23.1 (20.0–26.2)	43	7.7 (5.4–10.0)	<0.0001
IDU	58	2.3 (1.7–2.9)	54	2.9 (2.1–3.7)	4	0.4^§^ (0.1–0.7)	<0.0001
Stimulant use	197	8.5 (7.1–9.9)	178	10.2 (8.4–12.0)	19	2.9 (1.3–4.6)	<0.0001
Any opioid use	65	2.6 (1.9–3.2)	59	3.1 (2.3–3.9)	6	0.8^§^ (0.1–1.6)	0.002
**Receipt of services**							
Interpreter	420	18.9 (14.1–23.7)	283	16.4 (12.9–19.9)	137	27.1 (15.8–38.4)	0.0063
Transportation	550	24.5 (20.6–28.3)	364	21.4 (18.0–24.7)	186	34.8 (25.5–44.0)	0.0006
Meal	713	30.5 (27.4–33.7)	467	26.4 (23.4–29.4)	246	44.1 (39.4–48.8)	<0.0001
**Unmet need for services**							
Interpreter	23	0.8 (0.4–1.3)	16	0.8^§^ (0.3–1.3)	7	0.9^§^ (0.2–1.7)	0.7522
Transportation	224	9.4 (7.8–10.9)	165	9.2 (7.5–10.9)	59	10.0 (7.1–12.8)	0.5992
Meal	213	8.8 (7.5–10.2)	161	8.9 (7.4–10.3)	52	8.7 (6.2–11.2)	0.9237
**STD screening^¶^**							
Gonorrhea	1,334	54.1 (48.7–59.5)	1,020	55.0 (49.8–60.1)	314	51.2 (42.0–60.4)	0.3408
Chlamydia	1,325	53.9 (48.8–59.1)	1,013	54.7 (49.8–59.7)	312	51.2 (42.6–59.9)	0.3425
Syphilis	1,694	70.4 (67.2–73.7)	1,317	72.5 (69.1–75.8)	377	63.7 (57.3–70.2)	0.0069
**ART prescribed^¶^**	2,244	95.9 (95.1–96.7)	1,698	96.1 (95.2–97.0)	546	95.1 (93.2–97.1)	0.3652
Adherent, past 3 days**	1,939	88.5 (86.7–90.3)	1,461	88.1 (86.3–89.8)	478	90.1 (86.4–93.7)	0.3078
**Sustained viral suppression^¶^**	1,707	71.8 (69.4–74.2)	1,311	72.9 (70.2–75.6)	396	68.0 (62.7–73.3)	0.0888

**Abbreviations:** ART = antiretroviral therapy; CI = confidence interval; HIV = human immunodeficiency virus; IDU = injection drug use; STD = sexually transmitted disease.

* Numbers might not sum to total because of missing data. Percentages might not sum to 100 because of rounding. All estimates are based on self-report from interview except where otherwise noted. All estimates are based on the 12 months preceding interview except where otherwise noted.

^†^ Percentages are weighted percentages. 95% confidence intervals incorporate weighted percentages.

^§^ Coefficient of variation >0.30; estimate might be unstable.

^¶^ Estimates from medical record abstraction. Abstractions were performed at the usual source of outpatient HIV medical care in the 12 months before the last care visit.

** Among persons taking ART, took 100% of ART doses in the past 3 days.

## Discussion

Compared with men, more Hispanic/Latina women with HIV infection in care faced socioeconomic and language-related challenges than did men; however, they had similar prevalences of ART prescription and viral suppression. Hispanic/Latina women used ancillary services at higher rates than did Hispanic/Latino men, perhaps mitigating the effects of the noted challenges on their clinical outcomes.

The poverty rate among Hispanics or Latinos in the United States is approximately twice that of non-Hispanic whites, and women live in poverty at higher rates than do men (*4*). This study found that 78% of Hispanic/Latina women receiving HIV care lived at or below the federal household poverty level, compared with 54% of men. Poverty is known to affect management of HIV infection and is a paramount concern affecting all stages of the HIV care continuum (*5*). Some ART regimens require food; thus, lack of food might lead to nonadherence. Lack of transportation might pose barriers to attending medical appointments and obtaining medications. Women’s higher receipt of meal and transportation services might have helped alleviate the negative consequences of food insecurity and lack of transportation on their clinical outcomes.

Among racial and ethnic groups in the United States, Hispanics/Latinos are the group least likely to have any health insurance coverage (*6*). In this study, 22% of Hispanic/Latino men and 14% of Hispanic/Latina women had any private health insurance. However, 72% of Hispanic/Latina women and 54% of men relied on public insurance only. Taken together, 87% of women and 76% of men had some type of coverage. The higher coverage among women might also have contributed to similar clinical outcomes between men and women. Moreover, the Ryan White HIV/AIDS Program provides comprehensive care as well as support services for persons living with HIV infection who have no insurance or are underinsured and is associated with improved clinical outcomes among persons in poverty (*7*).

Overall, 38% of women and 21% of men reported not speaking English well, which can affect ability to understand a provider’s instructions and ability to navigate the health care system (*8*). In addition, the language barrier might prevent care providers from understanding the patient and could lead to missed opportunities to provide needed support or direction. Bilingual providers or interpreter services might have mitigated linguistic barriers.

Lower levels of substance abuse might also have contributed to better clinical outcomes among Hispanic/Latina women receiving HIV care. Persons who use drugs have been found to have lower levels of adherence (*9*) and, therefore, lower levels of sustained viral suppression, which is critical to reducing morbidity and mortality and preventing transmission to others.

Hispanics/Latinos in HIV care still have higher levels of unmet need for services when compared with other populations (*10*). Although no disparities between men and women in sustained viral suppression among Hispanics/Latinos were identified, levels are still lower than those found among non-Hispanic whites (*3*) and lower than the national prevention goal of at least 80% viral suppression for persons with diagnosed HIV infection.

Through partnerships that use a high-impact approach to advancing national HIV prevention goals, CDC works to improve health outcomes and reduce HIV transmission among all Americans. CDC provides support and assistance to health departments and community-based organizations deliver effective interventions to decrease HIV incidence among Hispanic/Latinos, improve their health outcomes, and reduce transmission. CDC also raises awareness about HIV among Hispanics/Latinos through Partnering and Communicating Together to Act Against AIDS (PACT),* which includes the National Hispanic Medical Association and is part of the larger Act Against AIDS initiative.

The findings in this report are subject to at least three limitations. First, the results might not be applicable to Hispanic/Latinos living with HIV infection who are not receiving medical care. Second, behavioral characteristics are self-reported and thus, might be subject to measurement error as well as reporting and social desirability biases. Finally, data were adjusted to minimize nonresponse bias based on known characteristics of sampled facilities and patients; however, the possibility of residual nonresponse bias exists.

Hispanic/Latino men and women with HIV-infection in care differ from one another in their behavioral and sociodemographic characteristics. Hispanic/Latina women receiving HIV care face more socioeconomic and language-related challenges than do men. However, rates of ART prescription and sustained viral suppression did not differ between Hispanic/Latino men and women, perhaps reflecting Hispanic/Latina women’s greater use of ancillary services. It is important for providers to be cognizant of the challenges faced by this population and assist with access to needed ancillary services. Although the lack of disparity in viral suppression among Hispanic/Latino men and women in HIV care is encouraging, work still remains to decrease ethnic disparities and attain national prevention goals among this population.

**Summary**

**What is already known about this topic?**

The prevalence of diagnosed human immunodeficiency virus (HIV) infection among Hispanics/Latinos in the United States is approximately twice that of non-Hispanic whites. Describing Hispanics/Latinos with HIV-infection in medical care by sex could inform service delivery.

**What is added by this report?**

During 2013–2014, among Hispanics/Latinos with HIV infection in care, women were significantly more likely than were men to live in poverty, have English language difficulties, and receive ancillary services. Prescription of antiretroviral therapy and sustained viral suppression did not significantly differ by sex.

**What are the implications for public health practice?**

Providers should be cognizant of the challenges faced by Hispanics/Latinos with HIV-infection in care and provide referrals to needed ancillary services.

## Footnotes

* https://www.cdc.gov/actagainstaids/partnerships/pact.html.
